# Propagation and Scattering of Lamb Waves at Conical Points in Plates

**DOI:** 10.1038/s41598-019-51187-9

**Published:** 2019-10-23

**Authors:** David M. Stobbe, Clemens M. Grünsteidl, Todd W. Murray

**Affiliations:** 1 Department of Mechanical Engineering, University of Colorado Boulder, Boulder, CO 80309 USA; 2 Research Center for Non Destructive Testing GmbH, 4040 Linz, Austria

**Keywords:** Mechanical engineering, Acoustics

## Abstract

Lamb waves exhibit conical dispersion at zero wave number when an accidental degeneracy occurs between thickness mode longitudinal and shear resonances of the same symmetry. Here we investigate the propagation of Lamb waves generated at the conical point frequency and the interaction of these waves with defects and interfaces. The group velocity and mode shapes of Lamb waves at the conical point are found, and it is shown that as the wavenumber gets close to zero, considerable group velocity is seen only for material properties supporting a degeneracy or near-degeneracy. The unusual wave propagation and mode conversion of Lamb waves generated at the conical point are elucidated through numerical simulations. Experimental measurements of near conical point Lamb wave interaction with holes in a plate demonstrate that these waves flow around defects while maintaining a constant phase of oscillation across that plate surface.

## Introduction

Lamb waves are guided waves that propagate through plates and are the result of reflection, refraction, and mode conversion of bulk waves from the plate surfaces. The theory of Lamb wave propagation is well established and Lamb waves have been used for a multitude of applications including nondestructive evaluation (NDE) of plates, structural health monitoring, and sensing systems^1–3^. Lamb waves follow a complex dispersion relation described by the Rayleigh-Lamb equation, and the specific shape of the dispersion curves in a homogeneous, isotropic plate is dictated by the Poisson’s ratio^4^. For a given application, it is often advantageous to access specific Lamb wave modes with favorable propagation characteristics. For long-range inspection applications, for instance, Lamb waves with small group velocity dispersion are used in order to minimize wave packet spreading with propagation distance. For local measurements, one can excite Lamb waves at particular points on the dispersion curve where the group velocity goes to zero while the phase velocity remains finite. At such zero group velocity (ZGV) points, a strong and localized resonance can be excited and used, for example, to determine elastic properties, monitor adhesion, or track fatigue damage^5–16^.

ZGV points are common to both acoustic and optical waveguides and are the result of a repulsion between two modes near a wave vector (*k*) of zero that leads to backward wave propagation, or a wave propagation with counter-directed phase and group velocities, in one of the modes^17,18^. The ZGV point is located at the transition between backward and forward wave propagation. The repulsion between modes is the strongest when an accidental degeneracy occurs^19^. In the case of Lamb waves, this means that there is a coincidence between the frequencies of longitudinal and shear resonances of the same symmetry^20^. A salient feature associated with accidental degeneracy is that the dispersion curve passes linearly through *k* = 0, allowing for a peculiar type of wave which propagates with an infinite wavelength but retains a finite group velocity. This wave propagates away from a source without a phase advance, producing a temporal oscillation of the plate that is uniform across the surface. Such behavior, evident in homogeneous elastic and optical waveguides, has also seen widespread attention in acoustic and optical “zero index” or “near zero index” metamaterials where linear dispersion, or conical dispersion in a three-dimensional sense, is achieved through a combination of materials structure and induced degeneracy^21–27^.

Recently, it was recognized and experimentally demonstrated that a simple, homogenous, aluminum plate shows linear dispersion through *k* = 0 at a particular Poisson’s ratio where accidental degeneracy occurs^28,29^. Degeneracy was induced by adjusting the elastic properties of the plate over a small range through temperature change. It was also shown that waves excited in a plate near *k* = 0, referred to as conical point waves, exhibited angle independent mode conversion upon encountering a plate edge. Here, we derive the group velocity of conical point Lamb waves through implicit differentiation of the Rayleigh-Lamb equations, and provide the mode shapes as *k* approaches zero. We track the group velocity of modes at low *k* values and show that long wavelength Lamb waves can propagate with non-negligible group velocity only if the mechanical properties are such that degeneracy or near-degeneracy on a given mode occurs. We provide a computational and experimental study of near conical point Lamb wave interaction with holes and other discontinuities in a plate and find that the near conical point waves flow around defects while maintaining a constant phase of oscillation across that plate surface. Mode conversion at a hole interface is nearly spatially isotropic, regardless of the position of the excitation source. We propose that conical point and near-conical point Lamb waves provide a facile means of investigating the physics of “zero index” wave propagation. In addition, for specific materials that exhibit near-degeneracy of modes with like symmetry, they could find application in the nondestructive characterization of elastic waveguides.

## Background and Theory

### Conical dispersion: group velocity and mode shape

**Figure 1:**
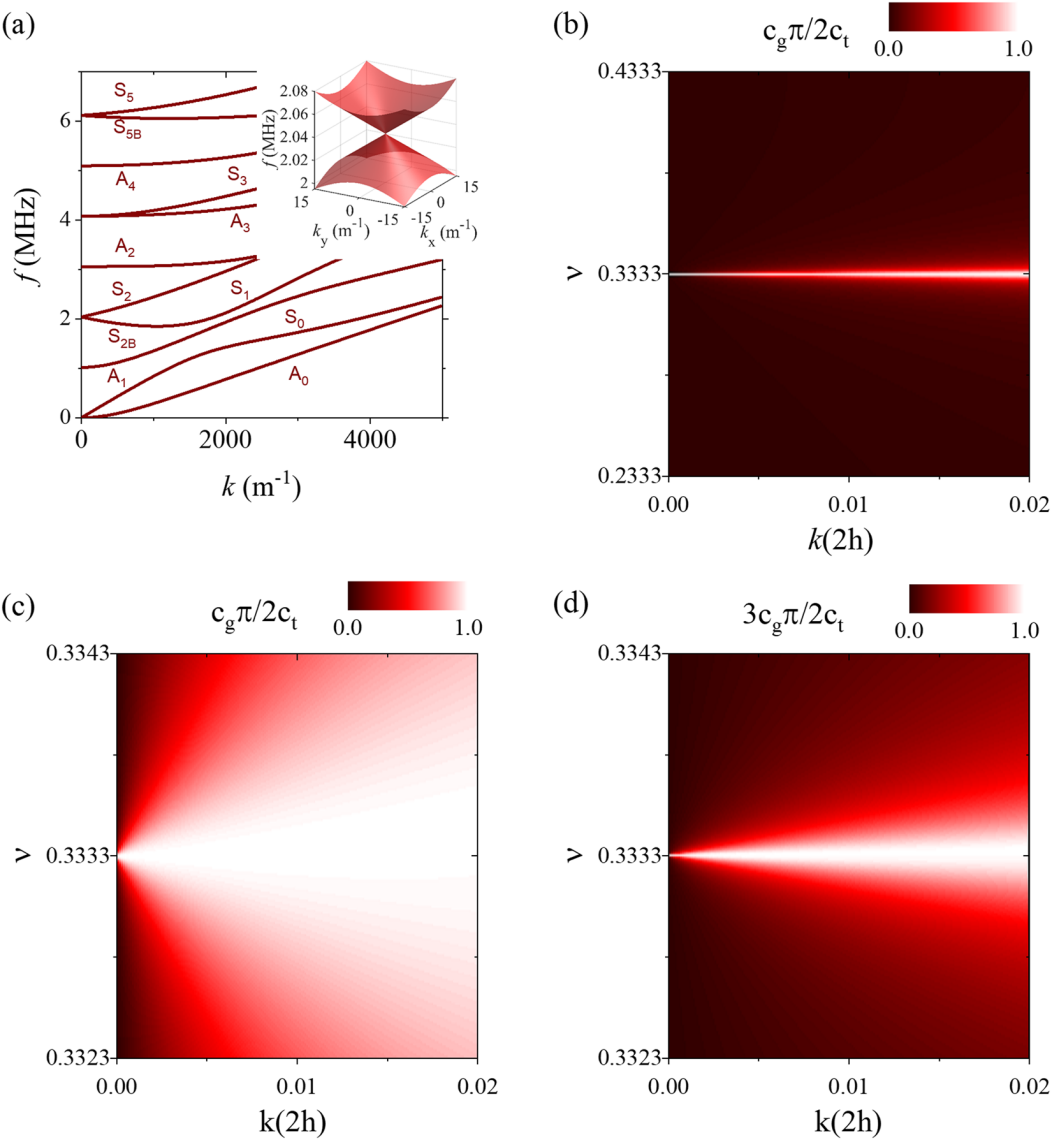
(**a**) Dispersion curve for an aluminum plate with a thickness of 1.54 mm, Poisson’s ratio of 1/3, and a longitudinal wave velocity of 627676 m/s. The inset shows a three-dimensional representation of the dispersion curve at the first conical point at 2.05 MHz, (**b**) the group velocity as a function of k(2 h) and Poisson’s ratio for the S_2_/S_2B_ mode and (**c**) a zoom in of the plot near the Poisson’s ratio that induces a degeneracy. (**d**) Similar plot obtained when tracking the group velocity of the S_5_/S_5B_ modes as a function of wave number and Poisson’s ratio.

A dispersion curve for a homogeneous, isotropic plate found using the Rayleigh-Lamb equations is shown in Fig. 1(a). Here, the longitudinal wave velocity c_1_ = 6276 m/s, the shear wave velocity c_2_ = 3138 m/s, and the plate thickness is taken as 1.54 mm. The plot shows both the antisymmetric (*A*_*i*_) and symmetric (*S*_*i*_) modes where the subscript represents the mode number. The modes are numbered with respect to the order which they occur with increasing temporal frequency on the *k* = 0 axis. Dispersion curves are generally parabolic at low wavenumber (*k*) values, and at the limit of *k* = 0 energy is trapped in the plate in the form of simple thickness resonances. Each resonance corresponds to a fundamental mode (longitudinal or shear) reflecting normally between the top and bottom faces of the plate. Such resonances occur at distinct frequencies determined by the plate thickness and longitudinal or shear wave velocity. In the special case of a plate in which the ratio of the longitudinal wave velocity (c_1_) and shear wave velocity (c_2_) is the ratio of two positive integers of different parity, then a longitudinal and a shear resonance of the same symmetry can occur at coincident frequencies. The interaction between the two bulk mode thickness resonances causes the otherwise stationary resonances to propagate at a finite group velocity, and results in linear dispersion at *k* = 0. For the dispersion curve in Fig. 1(a), for example, $\frac{{c}_{1}}{{c}_{2}}=2$ and degeneracies between modes of the same symmetry occur at 2.04 MHz and 6.12 MHz. Linear dispersion at *k* = 0 is also referred to as conical dispersion due to the fact that the dispersion surface in three dimensions forms the shape of a cone as shown in the inset of Fig. 1(a) for the lowest frequency degeneracy. At the conical point waves are produced which have an infinite wavelength (*k* = 0) and spread over the plate surface with a spatially uniform surface oscillation.

The group velocity ($\frac{d\omega }{dk}$) at the conical point was calculated by Mindlin by linearizing the Rayleigh-Lamb dispersion equations for small values of *k*^20^. Here we take an alternate, but essentially equivalent, approach to find the conical point group velocity. Considering symmetric modes only, the Rayleigh-Lamb frequency equation (${\varOmega }_{S}$) is given by:

1 $${\varOmega }_{S}(\omega ,k)=\frac{\tan (qh)}{\tan (ph)}+\frac{4{k}^{2}pq}{{({q}^{2}-{k}^{2})}^{2}}=0,$$

where ${p}^{2}={(\frac{\omega }{{c}_{1}})}^{2}-{k}^{2}$, ${q}^{2}={(\frac{\omega }{{c}_{2}})}^{2}-{k}^{2}$, *h* is half the plate thickness, and ω is the angular frequency. Given that $d\varOmega s(\omega ,k)=0$, the group velocity can be expressed as^30–32^:

2 $$\frac{d\omega }{dk}=-\frac{\partial {\varOmega }_{S}/\partial k}{\partial {\varOmega }_{S}/\partial \omega }.$$

We find the group velocity at a particular conical point by taking the limit of Eq. 2 for $k\to 0$. Here we consider, for example, the conical point that occurs at a frequency of $\omega =\pi {c}_{2}/h$ when ${c}_{1}=2\,{c}_{2}$. Taking the limit and applying L’Hôpital’s rule we find:

3 $$\mathop{\mathrm{lim}}\limits_{k\to 0}\frac{d\omega }{dk}=-\mathop{\mathrm{lim}}\limits_{k\to 0}\frac{\partial {\varOmega }_{S}/\partial k}{\partial {\varOmega }_{S}/\partial \omega }=-\mathop{\mathrm{lim}}\limits_{k\to 0}\frac{d/k(\partial {\varOmega }_{S}/\partial k)}{d/k(\partial {\varOmega }_{S}/\partial \omega )}=-\mathop{\mathrm{lim}}\limits_{k\to 0}\frac{{\partial }^{2}{\varOmega }_{S}/\partial {k}^{2}+{\partial }^{2}{\varOmega }_{S}/\partial k\partial \omega (\frac{d\omega }{dk})}{{\partial }^{2}{\varOmega }_{S}/\partial k\partial \omega +{\partial }^{2}{\varOmega }_{S}/\partial {\omega }^{2}(\frac{d\omega }{dk})}.$$

At *k* = 0, we find that ${\partial }^{2}{\varOmega }_{S}/\partial k\partial \omega =0$. Plugging this in and solving for the group velocity we obtain:

4 $${\frac{d\omega }{dk}}_{k=0}={(\frac{{\partial }^{2}{\varOmega }_{S}/\partial {k}^{2}}{{\partial }^{2}{\varOmega }_{S}/\partial {\omega }^{2}})}_{k=0}^{\frac{1}{2}}=\pm \frac{2{c}_{2}}{\pi }.$$

Here we assume the limit is unique regardless of the direction of the derivative. An identical result has been obtained by Delph *et al*. using a Taylor expansion of the characteristic equation^33^.

We now consider the mode shapes of plate oscillations at the conical point. Taking the case of plane strain where the waves propagate in the *x* direction and the *y* direction is perpendicular to the plate surface, the displacement fields associated with symmetric waves are given by^34^:

5 $$\begin{array}{rcl}{u}_{x} & = & i({\rm{A}}k\,\cos \,py+{\rm{B}}q\,\cos \,qy){{\rm{e}}}^{{\rm{i}}(kx-\omega t)}\\ {u}_{y} & = & (-{\rm{A}}\,p\,\sin \,py+{\rm{B}}k\,\sin \,qy){{\rm{e}}}^{{\rm{i}}(kx-\omega t)},\end{array}$$

where *A* and *B* are amplitude coefficients. The amplitude ratio can be found by applying traction free boundary conditions on the plate surfaces:

6 $$\frac{A}{B}=\frac{-2kq\,\cos \,qh}{(k^{2}-q^{2})\,\cos \,ph}$$

We again consider the case of ${c}_{1}=2\,{c}_{2}$ with linear dispersion near the conical point of the form $\omega (k)=\pi {c}_{2}/h\pm 2{c}_{2}k/\pi$. Substituting this into Eq. 5 and taking the limit as *k* approaches zero we find an amplitude ratio of $\frac{A}{B}=\pm 2$ and considering the real part of the displacement mode shapes in Eq. 4, the displacements near the conical point are:

7 $$\begin{array}{rcl}{u}_{x} & = & \frac{A\omega }{{c}_{1}}cos(\frac{\pi y}{h})\sin (\omega t)\\ {u}_{y} & = & -\frac{A\omega }{{c}_{1}}sin(\frac{\pi y}{2h})\cos (\omega t).\end{array}$$

The mode shape as *k* approaches the conical point is therefore the sum of two simple thickness resonances that oscillate out of phase and, interestingly, have equal amplitudes.

### Long wavelength Lamb waves

The existence of conical point dispersion in plates leads to a unique type of wave propagation. At *k* = 0 this is particularly evident; waves with infinite wavelength propagate through the plate with finite group velocity. A source exciting waves at the conical point thus produces a spatially uniform oscillation that spreads or flows over the plate surface and is insensitive to wavelength dependent wave phenomena such as diffraction and scattering. The connection between the temporal and spatial aspects of the wave field essentially breaks down. Here we examine the group velocity of long wavelength Lamb waves as a function of Poisson’s ratio (ν). The mode that originates at the longitudinal resonance frequency of $\frac{{c}_{1}\pi }{2h}$, corresponding to the S_2_ mode for $\nu  > \frac{1}{3}$ and the S_2B_ mode for $\nu  < \frac{1}{3}$, is first considered. At each value of Poisson’s ratio, Eqs 1 and 2 are solved to calculate the group velocity for $0 < k(2h) < 0.02$. The resulting plot is shown in Fig. 1(b), where the color scale gives the magnitude of the group velocity normalized to the conical point group velocity. A non-negligible group velocity is *only observed* in the vicinity of $\nu =\frac{1}{3}$ where the S_2_/S_2B_ mode accidental degeneracy leads to conical dispersion. The velocity is near the conical point velocity $({c}_{g}=\frac{2{c}_{2}}{\pi })$ for the degenerate case over this narrow range of *k* values due to the linear dispersion. Figure 1(c) shows a zoomed in region of the plot near $\nu =\frac{1}{3}$. Even in the case of near-degeneracy where the group velocity is zero at *k* = 0, exceptionally long wavelength Lamb waves with appreciable group velocity exist. From a practical standpoint, this gives some flexibility in materials selection in the experimental study of conical dispersion.

Figure 1(d) shows a similar plot for the mode that originates at the longitudinal resonance frequency of $\frac{3{c}_{1}\pi }{2h}$, corresponding to the S_5_/S_5B_ modes shown in Fig. 1(a). Here the degeneracy between the 3^rd^ longitudinal symmetric resonance and the 6^th^ transverse symmetric resonance occurs at $\nu =\frac{1}{3}$ and the conical point group velocity is ${c}_{g}=\frac{2{c}_{2}}{3\pi }$. Again, long wavelength Lamb waves with non-negligible group velocity are observed only near the degeneracy. Comparing the result with Fig. 1(c), the range of Poisson’s ratio over which the group velocity is enhanced is significantly narrower. This trend continues for higher order degeneracies.

## Experimental Set-Up

**Figure 2:**
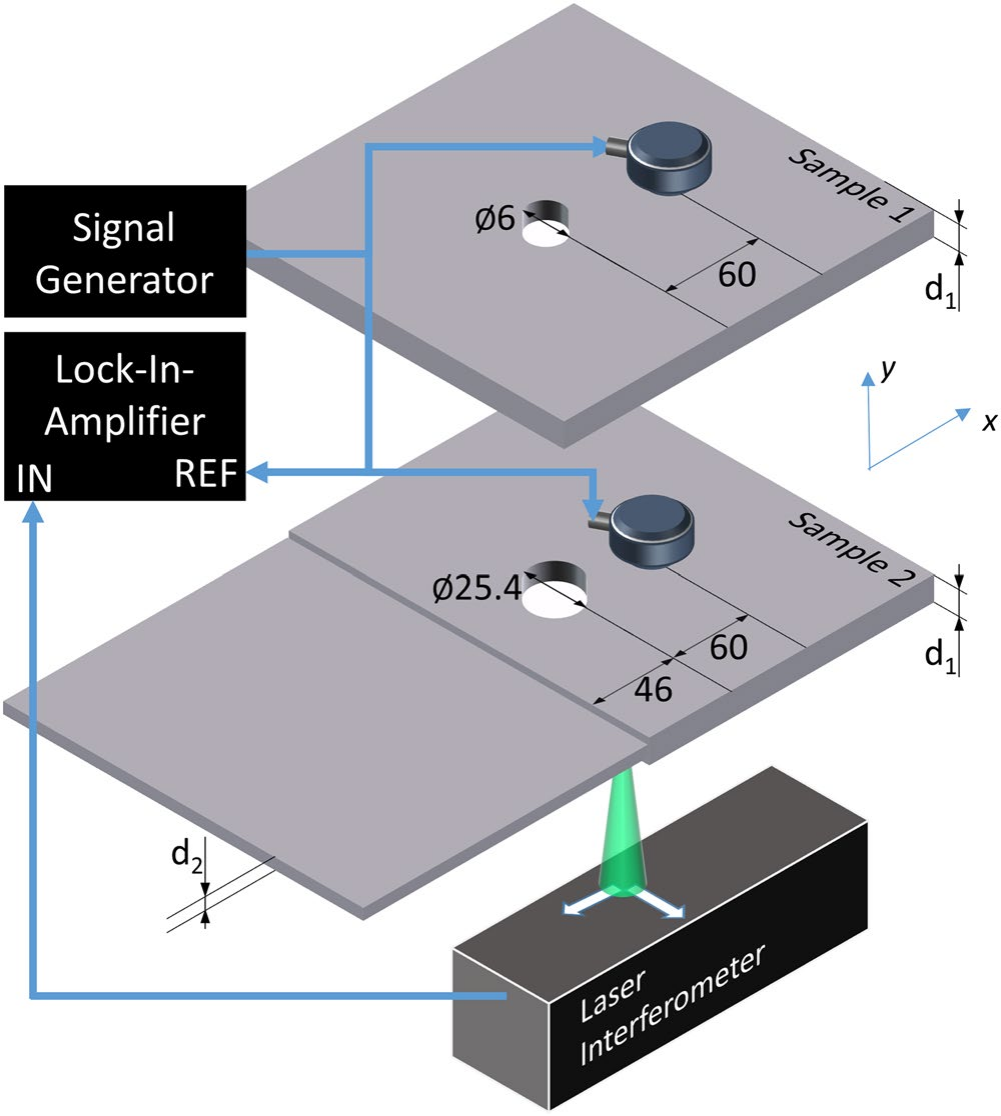
Experimental configuration for the generation and detection of conical point Lamb waves. A contact longitudinal mode transducer is coupled to the sample and driven with a continuous sine wave. The normal displacement is measured on the bottom surface of the sample using a photorefractive interferometer. Sample 1 has a thickness (d_1_) of 1.54 mm and Sample 2 has thicknesses of (d_1_) 1.54 mm and (d_2_) 1.45 mm.

A schematic of the experimental setup is shown in Fig. 2. Lamb waves were generated using a contact piezoelectric transducer (Olympus v109) with a diameter of 25.4 mm coupled to the plate surface using a thin layer of oil. The transducer was driven by a sinusoidal voltage from a function generator coupled to a power amplifier. The displacement of the plate surface was measured on the opposite side of the plate from the excitation source using a photorefractive crystal based interferometer^35^ incorporating a single-longitudinal mode frequency doubled Nd:YAG laser (λ= 532 nm) with an output power of 200 mW. The magnitude and phase of the plate displacement response at the excitation frequency were measured using an RF lock-in amplifier. The reference signal for lock-in detection was derived from the function generator driving the piezoelectric transducer. The lock-in time constant was 300 ms with a 12 dB per octave roll off. The detection point was scanned over the plate surface using a 2-axis computer controlled translation stage.

Experimental measurements were taken on the two sample geometries shown in Fig. 2, each using 6061-O aluminum alloy plates. The room temperature Poisson’s ratio of this alloy was found to be 0.3349 ± 0.0006 using the method developed by Clorennec *et al*. based on ZGV frequency measurements^8^. Further details on our measurement approach are available in the literature^29^. In sample 1, the transducer was placed 60 mm from a 6 mm diameter hole in a ${d}_{1}=1.54$ mm thick plate. The detection laser was scanned over a 40 × 40 mm grid surrounding the hole with a step size of 0.5 mm. In sample 2, a symmetric step change in plate thickness from ${d}_{1}=1.54$ mm to ${d}_{2}=1.45$ mm was created by masking the plate and immersing it in an acid bath. A 25.4 mm hole was machined in the thick side of the plate approximately 46 mm from the thickness step. The piezoelectric transducer was positioned 60 mm from the hole on the side opposite the step. The detection laser was scanned in a 130 × 80 mm (*x, y*) rectangular grid around the hole and across the thickness step using 0.5 mm steps. For both samples, thick adhesive tape was placed around the sample boundaries to help reduce the amplitude of edge reflections.

## Results and Discussion

In order to investigate conical point Lamb wave scattering, we first simulated Lamb wave interaction with a hole in a plate using a commercial finite element time domain software package (On-Scale, PZ-Flex). For the simulation, c_1_ = 6.00 mm/µs, c_2_ = 3.00 mm/µs $(\nu =\frac{1}{3}),$ and the plate thickness was 1.5 mm. The plate was modeled on an orthogonal grid with element dimensions 50 × 25 × 50 µm^3^ (*x*, *y*, *z*) and a 6 mm diameter hole was positioned in the center of the plate. Absorbing boundary conditions were used on the plate edges. Lamb waves were excited using a normal forcing function line source with a Gaussian spatial distribution normal to the line and a full width at half maximum (*FWHM*) of 12 mm. The source was 35 mm from the hole and temporally modulated at a frequency of 2.0 MHz, which corresponds to the frequency of the S_2_/S_2B_ conical point.

**Figure 3:**
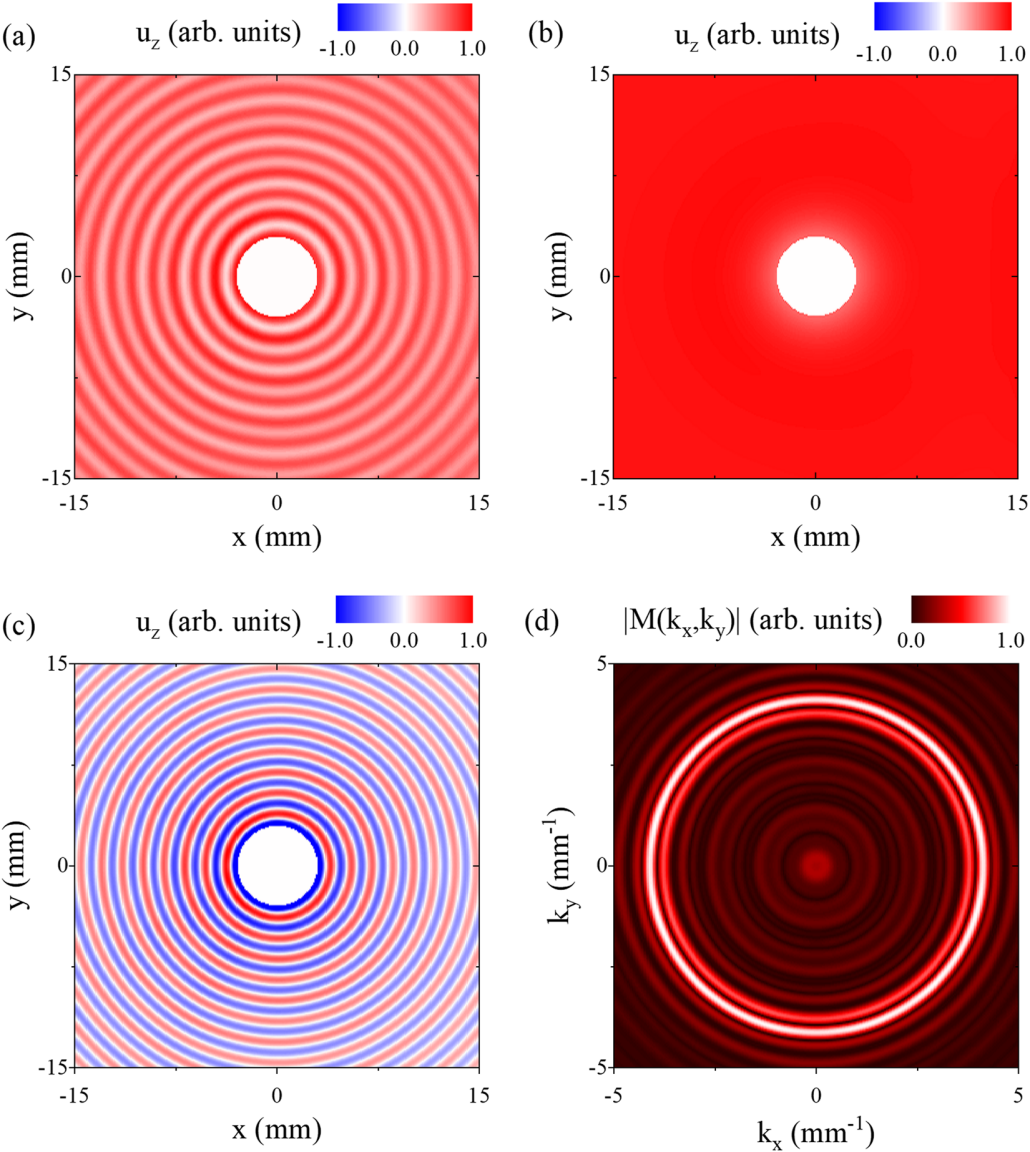
Finite element time domain simulation of conical point Lamb wave scattering from a hole. (**a**) Normal displacement field around the hole at steady state. The incident conical point mode creates an offset to the whole field and the higher wave number S_0_ mode stems from mode conversion at the hole boundary. (**b**) Low pass spatial filtered (k = 0.5 mm^−1^) displacement field to isolate the conical point mode. (**c**) High pass spatial filtered (k = 3.0 mm^−1^) displacement field to isolate the S_0_ mode which arises from mode conversion of the conical point mode at the hole boundary. (**d**) Magnitude of the FFT of the scattered field shown in (**c**).

The displacement normal to the plate surface is shown in Fig. 3(a). The field is displayed at after steady state has been reached at a time of 38 μs. In the figure, the waves are incident on the hole from the left side (negative x). The result shows a nearly perfectly symmetric periodic wave field surrounding the hole. Furthermore, the displacement field is offset from zero. Figure 3(b) shows the displacement field after processing with a spatial low pass filter with a cutoff of *k* = 0.5 mm^−1^ in order to isolate the conical point S_2_ mode. Remarkably, the conical point wave produces a spatially uniform oscillation of the plate surface that shows little evidence of perturbation by the hole.

Figure 3(c) shows the displacement field in Fig. 3(a) after a high pass spatial filter with a cutoff of *k* = 3.0 mm^−1^ in order to isolate the higher spatial frequency mode. This corresponds to the S_o_ mode which has a wave number of *k* = 4.14 mm^−1^. Here the spatially broad line source couples primarily into the long wavelength conical point S_2_ mode. At the free surface of the hole, the incident field is partially mode converted into the S_o_ mode in order to satisfy the traction free boundary conditions. Because the incident wave uniformly drives the mode conversion process around the hole, the S_o_ mode is emitted in phase around the hole producing the observed circular pattern. Figure 3(d) shows the magnitude of the two-dimensional Fourier transform of the displacement field in Fig. 3(c). The bright ring corresponding to the S_o_ mode ($|k\,|=4.14\,{{\rm{mm}}}^{-1}$) shows no evidence of directional dependence. This further demonstrates that the conical point wave is immune to scattering, producing a mode converted field of equal magnitude and phase over all points on the hole surface. We note that this also implies that the displacement field produced by the line source is independent of the position of the line with respect to the hole.

**Figure 4:**
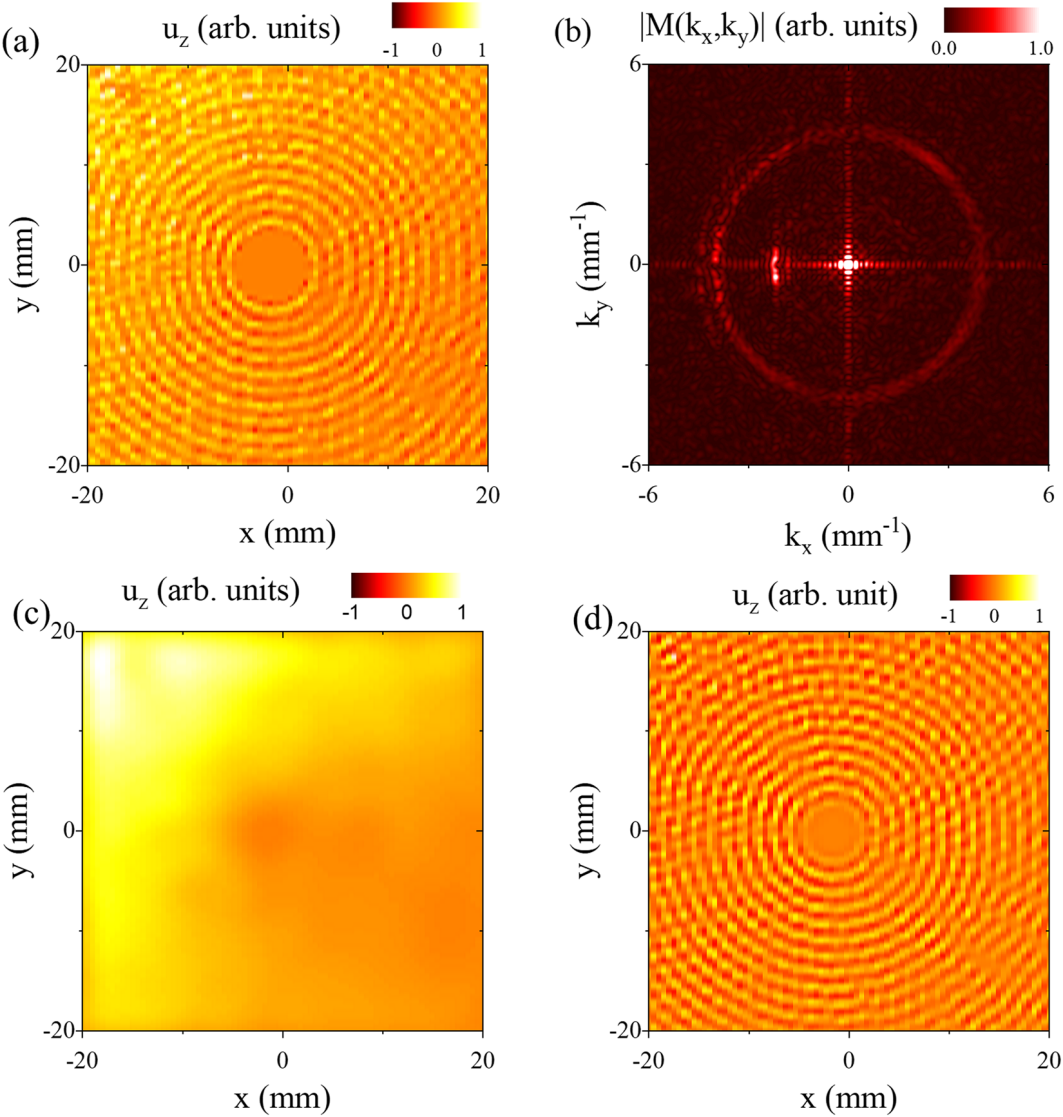
Measured normal displacement field of a conical point Lamb wave incident on a hole in the plate. (**a**) The measured displacement field is dominated by two modes, the incident conical point mode (DC component) and the scattered S_0_ mode (AC component). (**b**) Magnitude of spatial FFT of measured displacement field. The conical point mode appears as a bright spot near the origin and the scattered S_0_ mode appears as a circular ring of radius k = 4.0 mm^−1^. (**c**) Low pass filter (k = 0.8 mm^−1^) of measured displacement field to isolate the conical point mode. (**d**) Bandpass spatial filter (k = 3.8 mm^−1^ to k = 4.2 mm^−1^) to isolate the S_0_ mode.

Experimental measurements taken on sample 1 are shown in Fig. 4. As the Poisson’s ratio of the plate is slightly above $\frac{1}{3}$, we have a near-degeneracy and very long wavelength Lamb waves with non-negligible group velocity can be accessed. We choose an excitation frequency of 2.047 MHz, very close to the S_2_/S_2B_ (k = 0) cutoff frequencies. The magnitude and phase of the displacement field are measured at each location on the surface, and the resulting displacement field at an arbitrary phase is shown in Fig. 4(a). In agreement with the simulation, we observe the circular waves emitted from the hole boundary in phase due to mode conversion of the near conical point Lamb wave to the S_o_ mode. In addition, the near conical point mode causes an offset over the entire field of view. The interplay between the overall plate oscillation and the waves emitted by the hole is evident in Supplementary Movie 1. Figure 4(b) shows the magnitude of the spatial Fourier transform of the displacement field. The incident near conical point S_2_ mode appears as a bright spot near the origin and the S_0_ mode appears as a relatively uniform ring near $|k|=4.0\,{{\rm{mm}}}^{-1}$. Note that the contact transducer also generates an incident S_1_ mode that is observed over a limited angular range around $|k|=2.2\,{{\rm{mm}}}^{-1}$. This is due to the fact that the transducer does not act as a perfect Gaussian source and edge effects lead to some coupling into higher spatial frequency modes. The near conical point mode is observed in Fig. 4(c) by processing the measured displacement field with a low pass filter (*k* = 0.8 mm^−1^). The displacement is nearly uniform over the inspection area, and spatial variation can be attributed to both the finite wavelength of the mode and some change in detection sensitivity across the scan region. The S_0_ mode is isolated by processing the displacement field with a bandpass filter between *k* = 3.8 mm^−1^ and *k* = 4.2 mm^−1^. The experimental result, shown in Fig. 4(d), compares favorably with simulation (Fig. 3(c)). The S_0_ mode is generated with uniform magnitude and phase over the boundary of the hole and there is no indication of diffraction or scattering of the near conical point mode.

**Figure 5:**
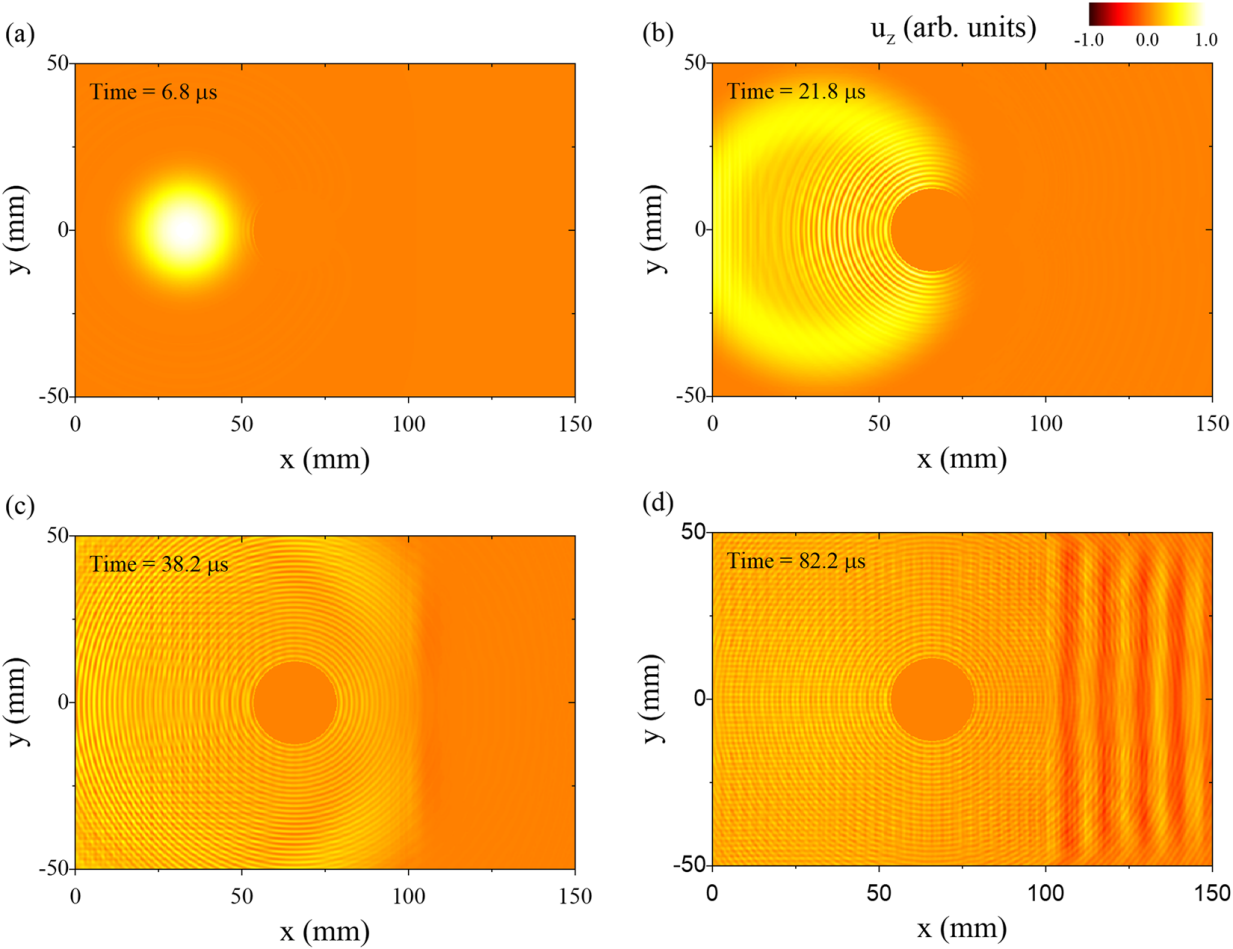
Numerical simulations of conical point Lamb waves interacting with a hole and a thickness step in a plate. (**a**) Displacement field prior to interaction with the hole. (**b**) Displacement field as the wave front begins impinging on the hole. (**c**) Displacement field after the wave-front has passed the hole. (**d**) Steady state displacement field. The mode converted S_2B_ mode after the thickness step, which originates mainly from the incident conical point mode, has planar wave-fronts despite the presence of the hole.

As a further demonstration of the unique nature of conical point waves, we now consider wave propagation in sample 2 (see Fig. 2). Here, the hole size is increased to 25.4 mm and the hole is positioned near a thickness step in the plate. We first consider a numerical simulation, where the plate is excited by a normal force with a Gaussian profile (FWHM = 25.4 mm) positioned 30 mm from the hole. The spot diameter was selected to match the size of the transducer used in the experiments. The hole is between the source and a symmetric thickness step, where the thickness changes from h_1_ = 1.50 mm to h_2_ = 1.48 mm (see Fig. 2). The mechanical properties of the plate and element grid size are the same as in the previous simulation, and the plate is excited with a sinusoidal forcing function at the conical point frequency of 2.0 MHz starting at t = 0. The evolution of the displacement field normal to the surface over time is shown in Fig. 5. At 6.8 μs, the conical point wave is spreading from the source and just begins to impinge upon the hole (Fig. 5(a)). The conical point mode then begins to envelop the hole, appearing to flow around the hole with uniform phase advance (Fig. 5(b)). There is little evidence of interaction of the conical point wave with the hole apart from mode conversion to the S_0_ mode at the interface. Figure 5(c) shows the displacement as the conical point mode is just incident on the step change in thickness at a time of 38.2 μs. At the interface, the conical point mode is converted primarily to the S_2B_ mode. Considering Snell’s law, the angle of refraction from a conical point mode at an interface would be zero degrees, regardless of the angle of incidence. This is evident in Fig. 5(d) where planar S_2B_ wave fronts are seen after the step. Note the uniformity of the S_2B_ mode and the absence of wave field perturbation by the hole. Higher order modes in the thin plate region produced by mode conversion of modes other than the conical point mode (such as the S_0_ mode) continue to diverge as expected.

**Figure 6:**
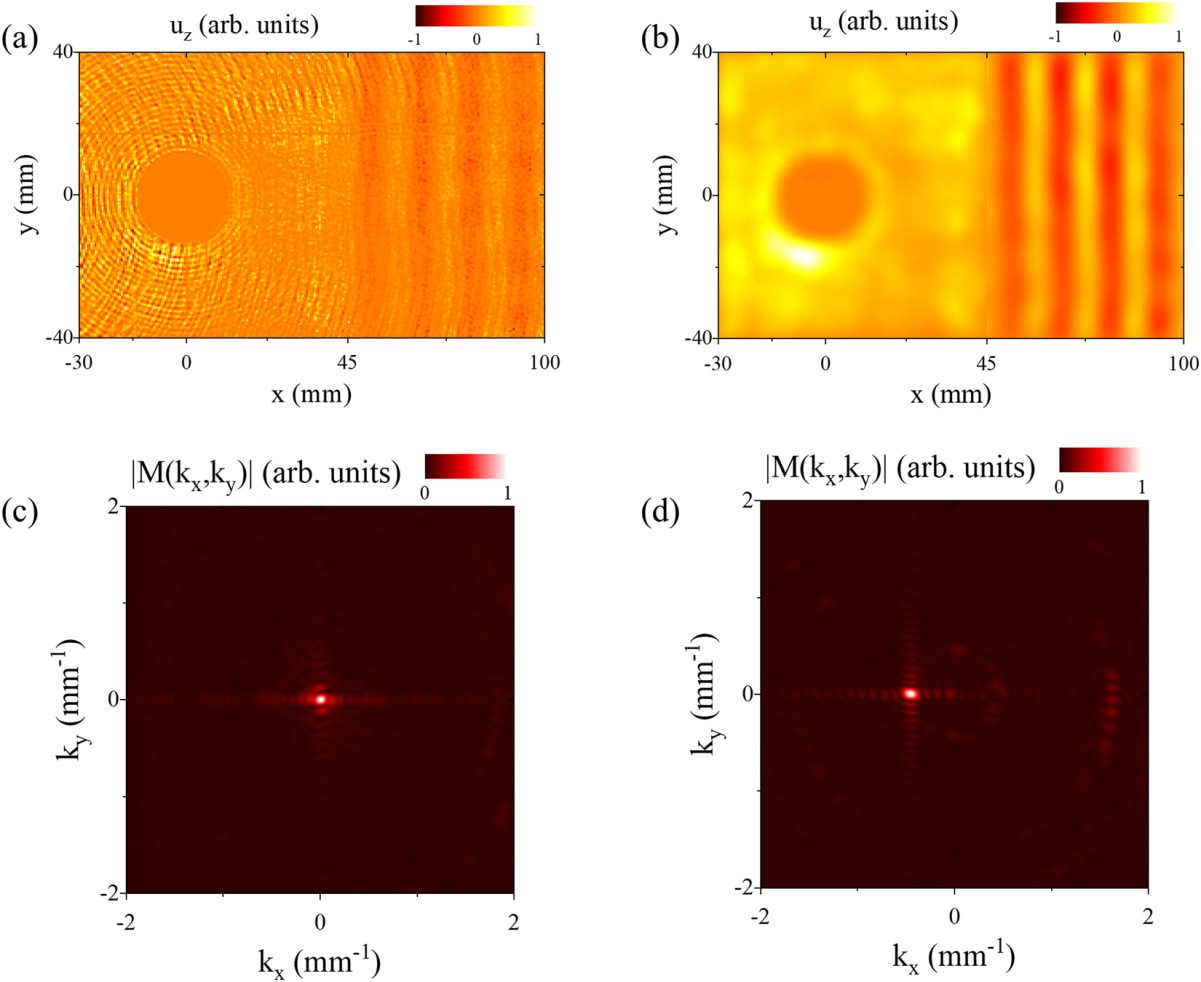
(**a**) Experimental result showing the steady state displacement field of the near conical point Lamb wave propagating past a hole to a thickness step in a plate. (**b**) Spatially filtered data to isolate the conical point mode at the left side of the interface (x < 45 mm) and the S_2B_ mode in the right side of the plate. (**c**) Magnitude of the spatial FFT of the raw data shown in (**a**) on the left side of the interface showing a strong response near k = 0. (**d**) Magnitude of spatial FFT of the raw data shown in (**a**) on the right side of the interface showing the S_2B_ mode over a very limited angular range.

The experimental measurements on sample 2 (see Fig. 2) are shown in Fig. 6. The plate was again excited near the S_2_/S_2B_ cutoff frequencies (k = 0) at 2.047 MHz. Figure 6(a) shows the displacement field measured across the plate surface. In agreement with the simulations, we observe mode conversion from the near conical point mode to the S_0_ mode at the hole boundary and to the S_2B_ mode at the interface (x = 45 mm). Figure 6(b) gives the filtered displacement field where on the left side of the step (x < 45 mm) a spatial low pass filter at *k* = 0.1 mm^−1^ is used to isolate the near conical point mode. On the right side of the step, a band pass filter between *k* = 0.3 mm^−1^ and *k* = 0.6 mm^−1^ is used to isolate the S_2B_ mode and only negative k values are retained to eliminate reflections from the plate edge. The spatial Fourier transforms of the unfiltered wave field before and after the step are given in Fig. 6(c,d), respectively. The near conical point mode (x < 45 mm) produces reasonably uniform oscillation of the plate close to *k* = 0. Beyond the interface, S_2B_ mode has a spatially uniform and planar phase front corresponding to the single bright spot on the Fourier transform near *k* = −0.5 mm^−1^. In agreement with the simulation, the uniformity of the S_2B_ mode is due to the absence of diffraction and scattering of the near conical point mode by the hole. Supplementary Movie 2 shows the propagation of the conical point mode and its conversion into plane S_2B_ backward waves.

## Conclusion

In conclusion, we have studied the propagation of conical point and near-conical point Lamb waves in an isotropic plate and the interaction of these waves with plate boundaries and interfaces. By calculating the group velocity of long wavelength Lamb waves as a function of Poisson’s ratio, we show that Lamb waves with non-negligible group velocities close to k = 0 exist only near degeneracies between thickness mode longitudinal and shear resonances of the same symmetry. Numerical simulations and experimental results demonstrate that Lamb waves generated at the conical point frequency are immune to scattering and diffraction from holes in the plate. Conical point waves appear to flow around holes and, in the steady state, the conical point mode produces a surface oscillation with a uniform phase over the plate surface irrespective of the presence of holes. Mode conversion of conical point waves to shorter wavelength modes at the free surface of the hole leads to a spatially isotropic wave field emitted perpendicular to the hole surface. Similarly, mode conversion of the conical point mode at a thickness step in the plate leads to a spatially uniform mode converted field propagating perpendicular to the step. In both cases, conical point modes exhibit an unusual spatial invariance where the steady state wave field is insensitive to the source location. Conical point dispersion in isotropic plates provides access to exceptionally long wavelength Lamb waves with high group velocity that may find application in nondestructive testing and Lamb wave based sensing systems.

## Supplementary information

Supplementary information

Supplementary Movie1

Supplementary Movie2
