# Supplementary material for: Propagation and Scattering of Lamb Waves at Conical Points in Plates
Source: Sci Rep. 2019 Oct 23;9:15216. doi: 10.1038/s41598-019-51187-9 (PMC6811593; doi:10.1038/s41598-019-51187-9)
Supplement: Supplementary file 1 — Supplementary information [file 41598_2019_51187_MOESM1_ESM.pdf]

Supplementary Information

Propagation and Scattering of Lamb Waves at Conical Points in Plates

David M. Stobbe[1], Clemens M. Grünsteidl[1,2], Todd W. Murray[1]

[1]*Department of Mechanical Engineering, University of Colorado at Boulder, Boulder, CO 80309*
[2]*Research Center for Non Destructive Testing GmbH, 4040 Linz, Austria*

Supplementary Movie 1

Movie of the measured surface displacement in sample 1. The left movie shows the raw data and the right movie shows the wave field after band-pass filtering to isolate the S_0 mode.

Supplementary Movie 2

Movie of the measured surface displacement in sample 2. The left movie shows the raw data and the right movie shows the wave field after band-pass filtering to isolate the conical point mode on the left side of the step and the S_{2B} mode on the right side.
