# Supplementary figures and images for: Propagation and Scattering of Lamb Waves at Conical Points in Plates
Source: Sci Rep. 2019 Oct 23;9:15216. doi: 10.1038/s41598-019-51187-9 (PMC6811593; doi:10.1038/s41598-019-51187-9)

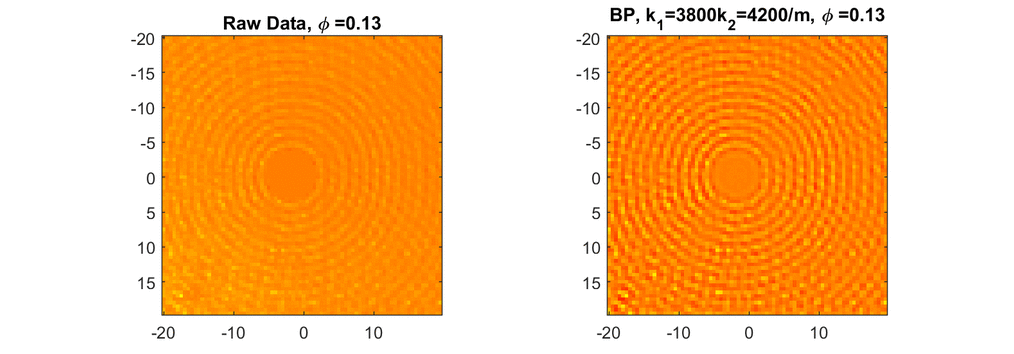

Supplement: Supplementary file 2 — Supplementary Movie1 [file 41598_2019_51187_MOESM2_ESM.gif]

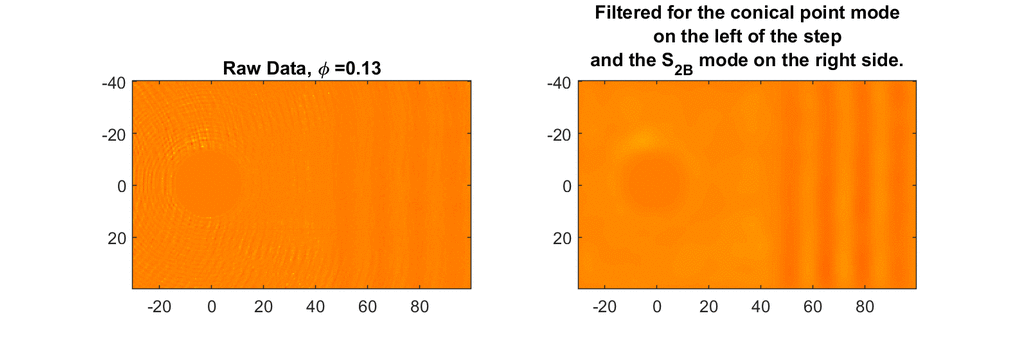

Supplement: Supplementary file 3 — Supplementary Movie2 [file 41598_2019_51187_MOESM3_ESM.gif]
